# Dissecting genomic hotspots underlying seed protein, oil, and sucrose content in an interspecific mapping population of soybean using high‐density linkage mapping

**DOI:** 10.1111/pbi.12929

**Published:** 2018-05-16

**Authors:** Gunvant Patil, Tri D. Vuong, Sandip Kale, Babu Valliyodan, Rupesh Deshmukh, Chengsong Zhu, Xiaolei Wu, Yonghe Bai, Dennis Yungbluth, Fang Lu, Siva Kumpatla, J. Grover Shannon, Rajeev K. Varshney, Henry T. Nguyen

**Affiliations:** ^1^ Division of Plant Sciences University of Missouri Columbia MO USA; ^2^ Center of Excellence in Genomics International Crops Research Institute for the Semi‐Arid Tropics Hyderabad India; ^3^ Division of Plant Sciences Laval University Laval QC Canada; ^4^ Crop Science Division Bayer CropScience Morrisville NC USA; ^5^ Dow AgroSciences Indianapolis IN USA; ^6^ Present address: Department of Agronomy and Plant Genetics University of Minnesota St. Paul MN 55108 USA; ^7^ Present address: Leibniz Institute of Plant Genetics and Crop Plant Research (IPK) Gatesleben D‐06466 Stadt Seeland Germany; ^8^ Present address: Nuseed Americas 10 N. East Street, Suite 101 Woodland CA 95776 USA; ^9^ Present address: Amgen One Amgen Center Drive Thousand Oaks CA 91320 USA

**Keywords:** soybean (*Glycine max*), seed composition traits, genomic hotspot, bin map, whole‐genome resequencing, quantitative trait loci, genomic‐wide association study

## Abstract

The cultivated [*Glycine max* (L) Merr.] and wild [*Glycine soja* Siebold & Zucc.] soybean species comprise wide variation in seed composition traits. Compared to wild soybean, cultivated soybean contains low protein, high oil, and high sucrose. In this study, an interspecific population was derived from a cross between *G. max* (Williams 82) and *G. soja* (PI 483460B). This recombinant inbred line (RIL) population of 188 lines was sequenced at 0.3× depth. Based on 91 342 single nucleotide polymorphisms (SNPs), recombination events in RILs were defined, and a high‐resolution bin map was developed (4070 bins). In addition to bin mapping, quantitative trait loci (QTL) analysis for protein, oil, and sucrose was performed using 3343 polymorphic SNPs (3K‐SNP), derived from Illumina Infinium BeadChip sequencing platform. The QTL regions from both platforms were compared, and a significant concordance was observed between bin and 3K‐SNP markers. Importantly, the bin map derived from next‐generation sequencing technology enhanced mapping resolution (from 1325 to 50 Kb). A total of five, nine, and four QTLs were identified for protein, oil, and sucrose content, respectively, and some of the QTLs coincided with soybean domestication‐related genomic loci. The major QTL for protein and oil were mapped on Chr. 20 (qPro_20) and suggested negative correlation between oil and protein. In terms of sucrose content, a novel and major QTL were identified on Chr. 8 (qSuc_08) and harbours putative genes involved in sugar transport. In addition, genome‐wide association using 91 342 SNPs confirmed the genomic loci derived from QTL mapping. A QTL‐based haplotype using whole‐genome resequencing of 106 diverse soybean lines identified unique allelic variation in wild soybean that could be utilized to widen the genetic base in cultivated soybean.

## Introduction

A major part of human, poultry, and livestock diets is derived from cereals and legumes (Mandal and Mandal, 2000). Legume seeds are sources of essential amino acids and minerals, and leguminous plants form a symbiotic relationship with rhizobia, providing biological nitrogen fixation (Gepts *et al*., 2005). Cultivated soybean [*Glycine max* (L.) Merr.] is a unique legume species that has 38%–42% protein, 18%–22% oil, and 4%–6% sucrose in seed and is commonly adapted to and grown in many areas in the world for human consumption, animal feed, and biodiesel production. Wild soybean species (*Glycine soja* Siebold & Zucc.), however, contains lower oil (8%–10%, nearly half of cultivated soybean), and lower sucrose (3%–4%), and relatively high protein content (46%–48%). Protein and oil contents in soybean seed are more important because approximately 60% of the value of soybeans comes from its protein meal, and the remaining 40% comes from its oil. In the United States, a minimum of 47.5% protein in soybean meal is demanded by the marketplace; however, the meal protein value of most commodity soybean cultivars is below this minimum threshold level (http://unitedsoybean.org/).

Steady gains have been made in soybean yield through breeding applications involving different approaches such as mutagenesis (Dobbels, 2017), quantitative trait loci (QTL) mapping (Warrington *et al*., 2015), and marker‐assisted selection (Patil *et al*., 2017a). The advanced breeding strategies mostly rely on a precise understanding of different aspects involved in the trait development. In soybean, the complex molecular and physiological mechanisms controlling yield, seed protein, and oil content are largely unknown (Patil *et al*., 2017a,2017b). Soybean seed composition is considered a complex and tightly regulated trait that is affected by the environment and environment × genotype interaction. Interdependency among seed composition traits results in strong negative correlations between different components, and with seed yield, makes it more challenging to increase any one of the seed composition trait (Bandillo *et al*., 2015; Chung *et al*., 2003; Kim *et al*., 2016). In addition, the differences in seed composition within the soybean germplasm are largely affected by genetic and epigenetic variation, expression profile of the genes involved in fatty acid biosynthesis, carbon partitioning, seed development, and possibly many other unknown regulators (Kim *et al*., 2016; Nichols *et al*., 2006; Sebolt *et al*., 2000). The genetic and molecular understanding of soybean seed protein and oil‐related traits will be helpful for the strategic development of improved soybean cultivars with optimized seed composition. The development of soybean with high oil and high protein would further increase the economic value of the crop by enriching the entire value chain from farmers to processors to end‐users.

The USDA soybean germplasm collection (http://www.ars-grin.gov/) currently preserves approximately 14 000 (*G. max*) and 1100 (*G. soja*) unique accessions with a wide range of genetic and phenotypic variation for seed composition traits. Cultivated soybean (*G. max*) that is evolved from wild soybean (*G. soja*) are closely related; therefore, the evolution of polymorphism at genomic and/or transcript level can be directly inferred (Patil *et al*., 2016; Valliyodan *et al*., 2016; Zhou *et al*., 2015). Wild soybeans (*G. soja*) germplasm with unique seed composition contrasting to the cultivated soybean provides relevant material for us to elucidate and understand molecular mechanism, evolution, and genetic regulation of the traits. The ability to produce interspecific hybridization between wild and cultivated soybeans makes it more valuable to explore the resources through molecular biology‐assisted breeding. However, the genetic resource of the wild species is still considerably untapped by soybean research community. Exploring the large collection of wild and cultivated soybeans and the functional analysis of naturally occurring genetic variation is one of the major frontiers in soybean protein, oil, and yield improvement.

Soybean seed composition is a quantitatively inherited trait controlled by multiple genes and regulators. In this regard, a large number of QTL for seed composition traits have been identified and genetically mapped with the advancement of genetic map construction (Hyten *et al*., 2010; Lee *et al*., 2015), the availability of a well‐annotated reference genome (Schmutz *et al*., 2010), resources for association mapping (Song *et al*., 2013, 2015), and whole‐genome resequencing data (Valliyodan *et al*., 2016; Zhou *et al*., 2015). These QTL were detected from varying genetic backgrounds and environments, using different genotyping, QTL mapping, and statistical methods. From previous studies, over 160 QTL for protein/oil and about 34 QTL for sucrose content have been reported in soybean (http//www.soybase.org). Among these, major QTL and genome‐wide association (GWA) loci for protein and oil content were consistently mapped on chromosome (Chr.) 20 (Chung *et al*., 2003; Diers *et al*., 1992; Nichols *et al*., 2006; Panthee *et al*., 2005; Pathan *et al*., 2013; Wang *et al*., 2015b; Warrington *et al*., 2015). There is, however, no report of identification of major and consistent QTL for sucrose content.

Recent advances in next‐generation sequencing technologies have provided a cost‐effective approach to develop several thousand single nucleotide polymorphisms (SNPs) in a limited period of time in large mapping populations, by sequence‐based genotyping (Kale *et al*., 2015). Whole‐genome resequencing (WGRS) of diverse germplasm or segregating recombinant inbred line (RIL) populations for targeted traits has shown to identify large number of SNPs and to pinpoint the genes associated with agronomic traits in several organisms (Huang *et al*., 2009a; Xu *et al*., 2012), including soybean (Qi *et al*., 2014; Xu *et al*., 2013). However, there has been a profound challenge when millions of SNPs that were developed by the WGRS method cannot be directly used for QTL mapping because it requires more computational resources to handle such large data sets (Asekova *et al*., 2016; Kale *et al*., 2015; Sonah *et al*., 2015). In addition, most of the SNPs segregate together as a haplotype, resulting in a high level of redundancy in the information. Use of a single SNP representing a haplotype or bin is sufficient to utilize the entire WGRS information for QTL mapping (Cheng *et al*., 2017; Patil *et al*., 2016). With the advent of bin mapping, candidate gene(s) were identified for plant height, grain width in rice (Huang *et al*., 2009a), and root‐knot nematode resistance and salt tolerance in soybean (Qi *et al*., 2014; Xu *et al*., 2013). In addition to bin mapping, SNP data obtained from WGRS can also be utilized for association mapping in a RIL population derived from biparental crosses (Sonah *et al*., 2013).

In this study, we used an interspecific mapping population developed from a cross between cv. ‘Williams 82’ and ‘PI 483460B’, a wild soybean accession. A combination of approaches [QTL mapping with 6K SNPs, bin mapping with skim‐WGRS data, genome‐wide association study (GWAS), and haplotype] was used to map novel alleles for seed composition trait QTL and to widen the genetic base in soybean towards the crop improvement. The objectives of this study were to identify QTL for protein, oil, and sucrose content and to determine additive/epistatic effects of the identified QTL in an effort to identify genes underlying these QTL.

## Results and discussion

### Phenotypic difference of protein, oil, and sucrose content

**Figure 1:**
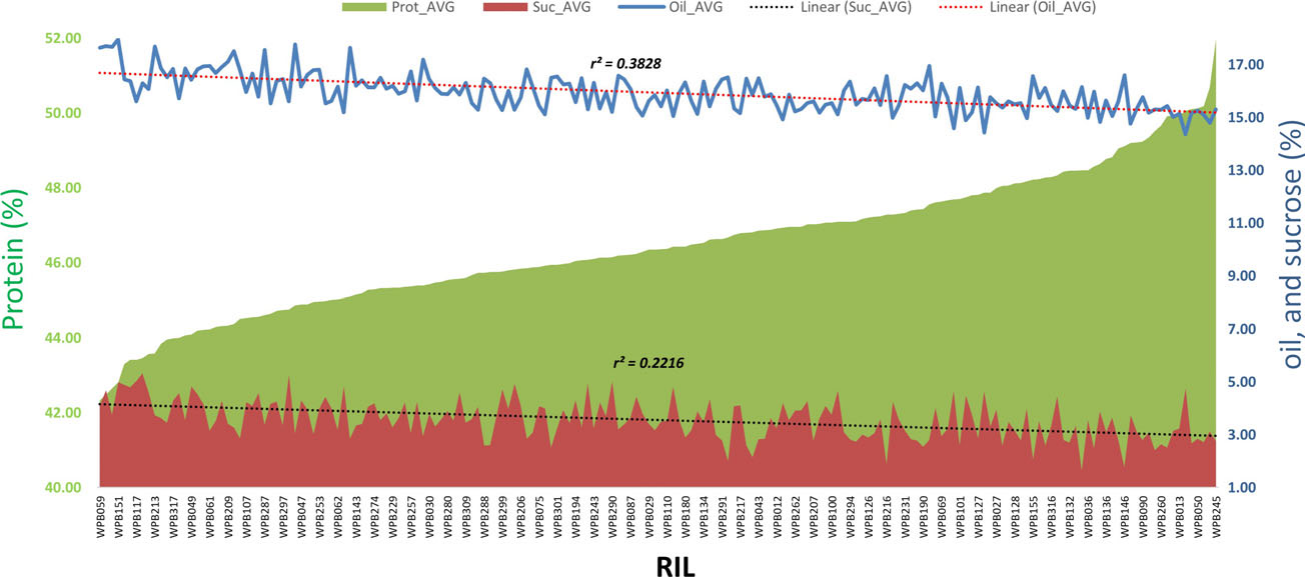
Pearson correlation coefficients between average protein, oil, and sucrose content in soybean seeds from the RIL population derived from Williams 82 × PI 483460B cross.

The parental genotypes, Williams 82 and PI 483460B, showed significant variation for all seed traits measured in this study (Table S1, Figure S1). The RILs showed a transgressive segregation where several RILs have exceeded in protein and sucrose content as compared to the parents, PI 483460B and Williams 82, respectively. However, no transgressive segregation was observed for oil content (Figure S1). The heritability of measured traits was calculated based on the analysis of variance of family means (Table S1). It is well studied that seed protein content is negatively correlated to seed oil and sucrose content in soybean (Nichols *et al*., 2006; Patil *et al*., 2017b; Sonah *et al*., 2015) Based on the phenotypic correlation among traits estimated for protein, oil, and sucrose across four environments, a similar trend was observed in this population (Figure 1). Moreover, the phenotypic data presented in this study supported the fact that soybean seed composition is significantly affected by environmental conditions (Bandillo *et al*., 2015; Chaudhary *et al*., 2015; Thomas *et al*., 2003).

The cost‐effective, accurate, and high‐throughput phenotyping for seed composition traits significantly contributed to the acceleration of genetic improvement through molecular breeding approaches. In soybean, major seed composition traits, for example protein, oil, and sucrose, are measured using high‐throughput near‐infrared reflectance (NIR) methods (Baianu *et al*., 2012). The HPLC (high‐performance liquid chromatography) platform is considered more reliable for sucrose content analysis due to its accuracy; however, this platform has limitations to use for high‐throughput assay and also involved higher costs per sample. Additionally, these wet‐laboratory techniques are associated with completeness of the extraction and the stability of the extracted compound during extraction steps, and hence, analysing these compounds in native matrix would be useful (Berardo *et al*., 2009; Brenna and Berardo, 2004; Delwiche *et al*., 2008). Considering these factors, a subset of 100 RILs was selected from the RIL mapping population. This subset was grown and harvested at the Bradford Research and Education Center (BREC), at the University of Missouri, Columbia, in 2013, and evaluated for sucrose content using the HPLC instrument. Correlation analysis was subsequently estimated for sucrose data between these two platforms (Figure S2). Although NIR instrument detected relatively higher sucrose content than HPLC platform, the trend showed a significant positive correlation (*r *=* *0.57**, *n* = 100). The results confirmed that NIR platform can generate reliable phenotypic data of soybean seed composition, which can be used for high‐throughput assessment of sucrose and for performing QTL analysis, screening diverse germplasm, and parental selection. Brenna and Berardo (2004) and Berardo *et al*. (2009) successfully utilized NIR spectroscopy for determination of seed composition traits including crude protein, crude lipid, starch, and carotenoids content in diverse maize germplasm. The composition data obtained from NIR were compared with HPLC using modified partial least‐squares equations, and it showed a high correlation between two platforms. In another study, Moncada *et al*. (2013) compared NIR and other techniques to determine antioxidant properties in quinoa. The results reported by the authors indicated that NIR provides an efficient and alternative method and the results are comparable with other platforms the chemical reference methods (Brenna and Berardo, 2004; Cozzolino, 2015; Moncada *et al*., 2013). Delwiche *et al*. (2008) developed the NIR equation and determined the potential of NIR to predict sucrose, glucose, and fructose content in mango with high precision. To confirm the reliability of NIR‐generated phenotypic data, we performed QTL analysis using these data sets that are reported in the other sections of the manuscript.

### Skim whole‐genome sequencing, SNP calling, and BIN map construction

**Figure 2:**
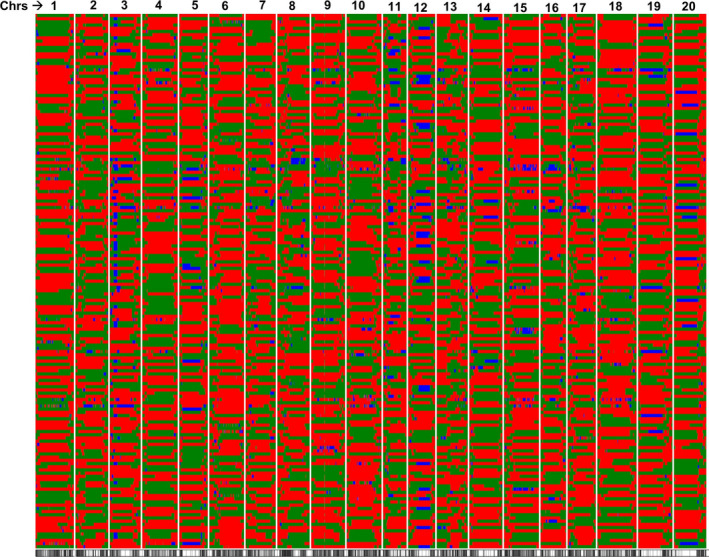
The recombination breakpoints identified in 188 RILs derived from Williams 82 × PI 483460B cross. The chromosomes are labelled as 1–20 and are separated by vertical lines while each horizontal line represents a single RIL. Green and red bars represent segments from Williams 82 and PI 483460B genotypes, respectively.

The RIL mapping population was genotyped using a skim sequencing approach (Golicz *et al*., 2015). The parental line, Williams 82, is the soybean reference genome (Schmutz *et al*., 2010), and to confirm the inferred genotypes, the parental line PI 483460B was sequenced at 15× genome coverage. A total of 1 410 571 SNPs were identified between PI 483460B and Williams 82 (W82.a2.v1) using SGSautoSNP pipeline (Lorenc *et al*., 2012). Subsequently, a total of 91 Gb of compressed Illumina paired‐end read sequence data were generated for 180 RILs with an average of 0.3× genome coverage. The identified SNPs from parental lines were compared with skim sequenced RIL population and 1 339 317 were found common between two sets. A total of 91 342 common SNPs after minor allele frequency (MAF) filtering (>0.20) were identified and considered for downstream analysis. The distribution of SNPs on the 20 soybean chromosomes (Chr.) is represented in Table S2 and Figure S3a. The sliding window approach (Huang *et al*., 2009b; Kale *et al*., 2015) was used on 91 342 SNP segregating in 180 RILs, and a total of 4070 bins were identified (Figure 2; Table S2). An average of 203 bins per chromosome was identified and mapped. The minimum number of bins (119) was identified on Chr. 11, whereas the highest number of bins (316) was identified on Chr.10 (Table S2).

### Linkage map construction with bin and BeadChip‐based SNP markers

A total of 4070 bins and BeadChips‐based 3343 SNP markers (hereafter referred to as 3K‐SNP) were used as molecular markers to construct two separate genetic linkage maps that spanned 2121 and 2925 cM, respectively. The order of bin markers on the genetic map was compared with their physical positions on the soybean reference genome sequence (W82.a2.v1) and a high concordance was observed for all the chromosomes (Figure S4).

### QTL analysis for seed protein, oil, and sucrose

**Figure 3:**
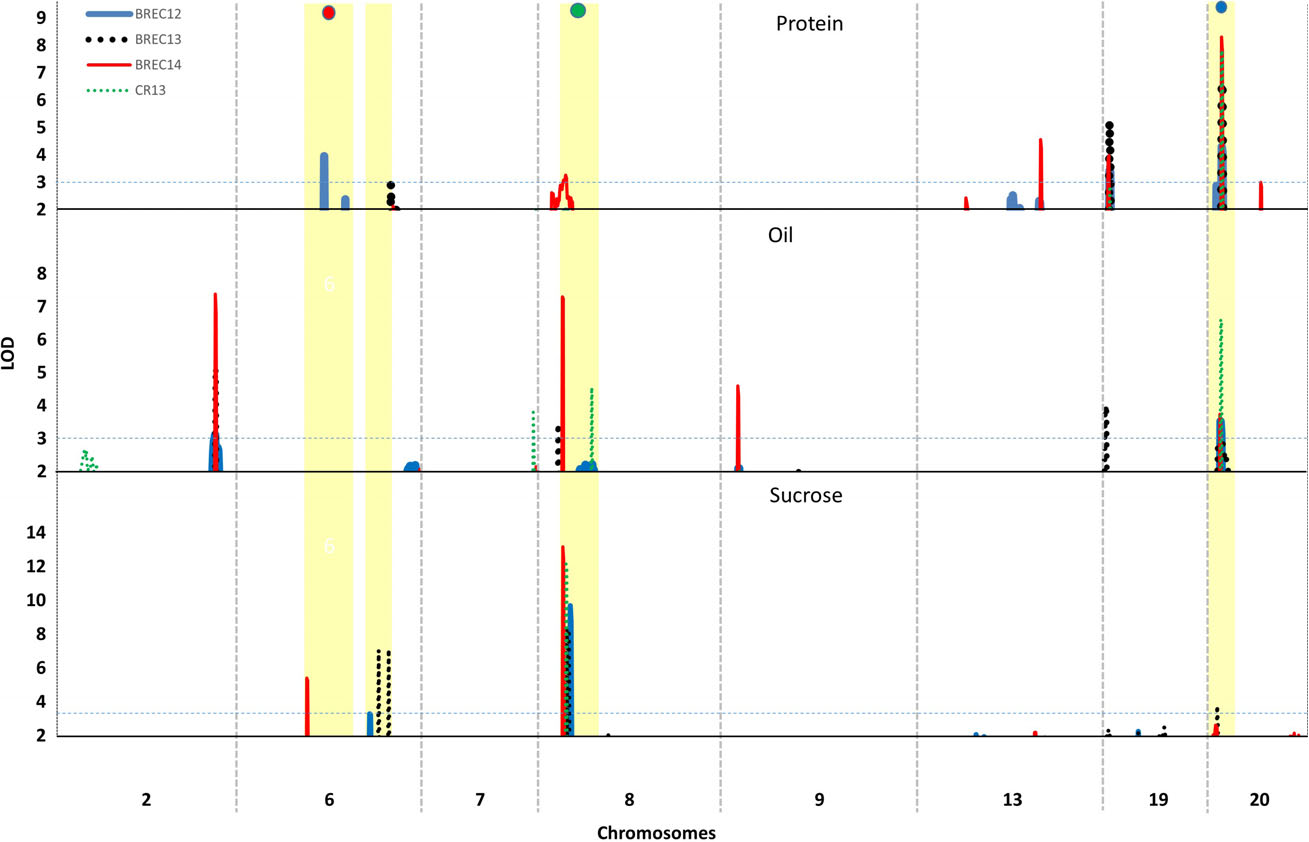
Genome‐wide distribution of quantitative trait loci (QTLs) identified for soybean seed protein, oil, and sucrose. QTL overlapped with domestication‐related genomic loci, maturity (*E1*,* E4*), and seed coat colour (*I* locus) are marked with red, purple, and green dots. Horizontal line on *Y* axis represents significant LOD score based on permutation test.

The genome‐wide permutation‐derived LOD score significance criterion for evaluating those observed QTL peaks scores was 2.9, 3.1, and 3.0 for declaring QTL significantly associated with seed protein, oil, and sucrose content, respectively. Composite interval mapping using bin markers identified five QTL for protein content, nine for oil content, and four for sucrose content (Table 1). The distribution of all major QTL in the soybean genome is shown in Figure 3 and summarized in Table 1.

**Table 1:** Comparative analysis of quantitative trait loci (QTLs) identified for protein, oil, and sucrose traits in the Williams 82 × PI483460B population using skim‐WGS and 3K SNP marker data sets

Trait	Environment	Skim_WGS								3K_Chip				
		QTL name	Marker interval (location)	Phy. interval (Kb)	Genetic interval (cM)	LOD	*R* ^2^	Add. effect	PVE (%)	QTL name	Maker interval	LOD	*R* ^2^	Phy. location (Mb)
Protein	BREC12	*qPro_06*	bin_6_21984752∼bin_6_22687378	702.62	0.31	3.9	9.7	−0.70	30.4	*qPro_06*	SNP8573_PI516C∼BARC_054471_12085	5.3	8.9	20.2–23.1
		*qPro_19*	bin_19_1024102∼bin_19_1420747	396.64	3.86	3.4	8.4	0.62		*qPro_19*	NCSB_004483∼NCSB_004480	4.2	7.6	18.4–20.3
		qPro_20	bin_20_2558940			2.9	6.9	0.57		*qPro_20*	NCSB_004739∼SNP30392_PI516C	4.1	6.5	2.50–3.2
		*qPro_20*	bin_20_33975596∼bin_20_34027051	51.41	0.32	2.9	5.4	0.50		*qPro_20*	BARC_027790_06672∼BARC_062091_17651	4.4	8	33.8–37.4
	BREC13	*qPro_19*	bin_19_1024102∼bin_19_1420747	396.64	3.86	5.2	10.7	0.70	24.7	*qPro_06*	BARC_047703_10385∼BARC_038923_07397	3.6	5	49.6–50.2
		*qPro_20*	bin_20_33975596∼bin_20_34027051	51.41	0.32	6.6	14	0.82		*qPro_15*	BARC_040373_07720∼SNP35548_Magellan	4.2	5.4	45.2–52.0
										*qPro_20*	NCSB_004738∼SNP30369_PI516C	5.0	6.2	22.2–25.6
										*qPro_20*	BARC_062091_17653∼BARC_027790_06672	6.9	15.8	33.8–37.4
	BREC14	*qPro_08*	bin_8_7951579∼bin_8_8602440	650.8	1.52	3.1	4.6	−0.46	32.7	*qPro_06*	BARC_047703_10385∼BARC_038923_07397	4.3	6.1	49.6–50.2
		*qPro_13*	bin_13_33162248∼bin_13_33151214	11.03	4.61	4.5	7.4	0.59		*qPro_20*	NCSB_004739∼SNP30392_PI516C	3.5	5	22.2–25.6
		*qPro_19*	bin_19_1024102∼bin_19_1420747	396.64	0.3	3.9	6.4	0.55						
		*qPro_20*	bin_20_33975596∼bin_20_34027051	51.41	0.32	8.3	14.3	0.83		*qPro_20*	BARC_062091_17652∼BARC_062091_17653	7.5	11.2	33.8–37.4
	CR13	*qPro_19*	bin_19_1147261∼bin_19_1420747	273.4	3.86	2.1	4.6	0.43	24.2	*qPro_05*	BARC_029787_06341∼BARC_029787_06360	4.3	6.3	
		*qPro_20*	bin_20_33975596∼bin_20_34027051	51.41	0.32	7.8	19.6	0.90		*qPro_15*	NCSB_003365∼NCSB_003366	6.3	9	
										*qPro_20*	NCSB_004738∼SNP30369_PI516C	4.5	5.6	
										*qPro_20*	BARC_064871_18952∼BARC_062091_17651	8.7	13.3	
Oil	BREC12	*qOil_02*	bin_2_45571767∼bin_2_45622096	50.32	0.61	3.1	7.1	0.17	23.7	*qOil_02*	BARC_028373_05856∼BARC_031227_07011	6.0	12.4	43.4–46.6
		*qOil_17*	bin_17_2091529∼bin_17_2097727	6.19	0.21	3.0	7.6	−0.17						
		*qOil_20*	bin_20_33975596∼bin_20_34027051	51.41	0.32	3.5	9	−0.19		*qOil_20*	BARC_027790_06672∼NCSB_004789	5.1	10.4	33.9–34.8
	BREC13	*qOil_02*	bin_2_45571767∼bin_2_45623045	51.27	0.31	5.1	10.1	0.27	35.9	*qOil_02*	SNP3239_PI516C∼SNP3244_PI516C	3.6	5.3	43.4–46.6
		*qOil_08*	bin_8_7542280∼bin_8_7564536	22.25	0.3	3.4	6.6	−0.22						
		*qOil_15*	bin_15_5286513∼bin_15_5384732	98.21	0.3	3.2	6.2	−0.21		*qOil_15*	BARC_040373_07720∼SNP35548_Magellan	3.6	5.2	4.56–5.20
		*qOil_19*	bin_19_1024102∼bin_19_930385	93.71	0.91	4.0	7.7	−0.24						
		*qOil_20*	bin_20_33975596∼bin_20_34027051	51.41	0.63	3.0	5.3	−0.20		*qOil_20*	BARC_062091_17653∼BARC_027790_06672	4.8	7.2	45.1–46.3
	BREC14	*qOil_02*	bin_2_45571767∼bin_2_45623045	51.27	0.31	7.3	13.5	0.25	41.4	*qOil_02*	SNP3244_PI516C∼NCSB_000498	7.2	10.5	43.4–46.6
		*qOil_08*	bin_8_7951579∼bin_8_8095904	144.32	0.3	7.2	13.3	−0.24		*qOil_08*	NCSB_001712∼SNP16086	8.3	12.3	7.99–8.10
		*qOil_09*	bin_9_5819186∼bin_9_5896549	77.36	0.31	4.6	8.1	0.19		*qOil_09*	BARC_060871_16939∼NCSB_002034	3.5	4.9	5.60–6.21
		*qOil_20*	bin_20_33975596∼bin_20_34027051	51.41	0.32	3.7	6.5	−0.17		*qOil_15*	BARC_039433_07497∼BARC_054257_12402	3.9	5.5	4.52–5.21
										*qOil_20*	BARC_062091_17651∼BARC_062091_17652	6.3	9.1	45.1–46.3
	CR13								32.4	*qOil_05*	NCSB_001051∼NCSB_001052	11.7	16.2	38.3–38.6
		*qOil_07*	bin_7_42136084∼bin_7_42040256	95.82	0.62	3.7	6.7	−0.30		*qOil_07*	SNP10717_PI516C∼BARC_028517_05936	4.5	5.8	43.5–43.7
		*qOil_08*	bin_8_11422070∼bin_8_11703885	281	0.92	4.5	8.1	−0.33		*qOil_08*	SNP15995–NCSB_001726	3.5	4.6	7.99–8.10
		*qOil_14*	bin_14_8471719∼bin_14_7775075	697	0.62	3.2	5.7	−0.28		*qOil_15*	NCSB_003365∼NCSB_003366	5.2	6.4	4.52–5.21
		*qOil_20*	bin_20_33975596∼bin_20_34027051	51.41	0.32	6.4	11.9	−0.40		*qOil_20*	BARC_062091_17653∼BARC_027790_06672	5.2	6.9	45.1–46.3
Sucrose	BREC12	*qSuc_06*	bin_6_37707727∼bin_6_38033477	325.75	2.43	3.3	7.4	0.22	31.8	*qOil_06*	NCSB_001275∼NCSB_001285	4.6	9.2	36.8–38.0
		*qSuc_08*	bin_8_9130096∼bin_8_9319041	188.94	0.3	9.7	24	0.38		*qSuc_08*	NCSB_001722∼BARC_035233_07151	9.8	22.6	9.04–9.39
	BREC13	*qSuc_06*	bin_6_40104066∼bin_6_41202830	1470	2.43	7.1	13	0.36	35.9	*qSuc_06*	BARC_061709_17355∼SNP30079	7.5	12.4	36.7–38.0
		*qSuc_08*	bin_8_8561674∼bin_8_8602440	40.76	0.3	8.3	16	0.38		*qSuc_08*	SNP16067∼BARC_010947_01738	11.9	20.4	9.04–9.39
		*qSuc_20*	bin_20_2558940∼bin_20_2386021	172.91	0.95	3.8	6.5	−0.25						
	BREC14	*qSuc_06*	bin_6_18870607∼bin_6_19091375	220.76	3	5.4	10	0.31	42.4	*qSuc_06*	SNP35899∼BARC_014203_02694	6.3	8.5	18.6–19.1
	CR13	*qSuc_08*	bin_8_7951579∼bin_8_8095904	144.32	0.3	13	27	0.49		*qSuc_08*	SNP16067∼BARC_010947_01738	17.4	27.5	9.04–9.39
		*qSuc_08*	bin_8_8561674∼bin_8_8602440	40.76	0.3	12	29	0.44	28.7	*qSuc_08*	SNP16086∼BARC_016683_03318	17.0	35.4	8.51–8.72

The physical location for bin markers is designated within the marker id.

For protein content, five QTL were mapped on Chrs. 6, 8, 13, 19, and 20. These QTL explained 4.6%–19.6% of the phenotypic variation for protein content with an average of 26.6% of total phenotypic variation across four environments (Table 1). The qPro_20 QTL accounted for all four environments (LOD of 2.97–8.3) followed by the next largest QTL, qPro_19 (LOD of 3.43–5.23). Comparison between 3K‐SNP and bin markers, the major qPro_20 QTL, was consistent in both the data sets for all four environments while qPro_06 and qPro_19 were consistent only in one environment (BREC12). The bin mapping identified more QTL than 3K‐SNP. A significant concordance was observed between bin and 3K‐SNP markers QTL based on physical location.

Since availability of genetic linkage map in soybean and over the last decade, more than 175 QTL from >39 independent studies have been reported for soybean seed protein and oil content (www.soybase.org). The comparative analysis among these QTL suggested that the major protein/oil QTL on Chr. 20 has been consistently mapped and a remarkable attention has been given to this QTL due to high additive effect (12%–55% phenotypic variation) and stability (Nichols *et al*., 2006; Patil *et al*., 2017b; Wang *et al*., 2015b; Warrington *et al*., 2015). In addition to QTL on Chr. 20, protein QTL on Chr. 6, Chr. 10, and Chr. 15 were identified in several mapping studies (Kim *et al*., 2016; Nichols *et al*., 2006); however, Chr. 20 (cqPro‐20) and Chr.15 (cqPro‐15) are designated as officially confirmed QTL by Soybean Genetics Committee (www.soybase.org) based on error rate and confirmation study showing alleles segregating in the test populations with different genetic backgrounds and maturity. However, most of the other reported QTL are not consistent due to genotype specificity or environment sensitivity which is also observed in our study. Recently, Phansak *et al*. (2016) performed selective genotyping of multiple biparental populations (48 F_2:3_) and identified significant QTL on one or more chromosomes including major QTL as discussed in our study. The large effect QTL alleles were identified from germplasm accession that would serve as parental donors in cultivar development programmes (Phansak *et al*., 2016). The Chr. 20 protein QTL mapped by Warrington *et al*. (2015) in the Benning × Danbaekkong population showed that a favourable allele from Danbaekkong imparts a total of 55% of the phenotypic variation and exerted little negative drag on seed yield in that population. However, in other studies, the presence of qPro_20 was negatively correlated with seed yield (Chung *et al*., 2003; Nichols *et al*., 2006; Sebolt *et al*., 2000), suggesting that Danbaekkong may have a unique allele than these sources or may have a genetic background that mitigates the yield drag of the QTL. Our recent study showed that the North American ancestors and elite cultivars carry different allele at qPro_20 as compared to *G. soja* and several high protein Korean accessions including Danbaekkong (Patil *et al*., 2017b). In this study, we further analysed the qPro_20 QTL for haplotype analysis using whole‐genome sequencing data and inferred the allelic variation within this QTL region.

For oil content, nine QTL were identified in four environments using bin linkage map. Among these, QTL on Chr. 2 (qOil_02), Chr. 8 (qOil_08), and Chr. 20 (qOil_20) were consistently detected and mapped in more than two environments. Interestingly, the qOil_20 with LOD values of 3.0–6.4 was genetically mapped to the same genomic location as the qPro_20. In agreement with earlier studies (Chung *et al*., 2003; Wang *et al*., 2015a,2015b), QTL on Chr. 20 exhibited pleiotropic effect on seed protein and oil content, thus resulting in a strong negative correlation between the two traits. It was noted that a favourable allele in this QTL region inherited from the wild soybean parent, PI 483460B. The qOil_02 identified in three environments and contributed by an allele from Williams 82, while many other QTL, such as qOil_07, qOil_09, qOil_14, qOil_15, qOil_17, and qOil_19, were detected in only one environment. Meanwhile, QTL, qOil_09, qOil_15, qOil_19, and qOil_20, were overlapped with similar region of previously reported QTL (Phansak *et al*., 2016; Qi *et al*., 2011; Wang *et al*., 2015a).

For seed sucrose content, four QTL were identified and mapped on Chrs. 6, 8, 16, and 20 which were designated qSuc_06, qSuc_08, qSuc_16, and qSuc_20, respectively (Table 1). Among these, the qSuc_08 was a major QTL for sucrose. These QTL were consistently detected across all four environments and showed high LOD values, ranging from 8.3 to 13.2 and explained 16.0%–29.0% of the total phenotypic variation. Similarly, the qSuc_06 identified as a second major QTL in three environments, except the Costa Rica nursery in 2013 (CR13), and explained 7.4%–13.4% phenotypic variation. Compared to the 3K‐SNP genetic map, an additional QTL for sucrose (qSuc_20) were identified using bin mapping, in which a favourable allele was inherited from the wild soybean parent, PI 483460B, with *R* ^2 ^= 6.5%. Further, we used a subset of 100 samples, phenotyped using HPLC and NIR platforms for QTL analysis. We identified a significant and consistent QTL qSuc_06 using both platforms suggesting that qSuc_06 could be a promising sucrose QTL. The second QTL qSuc_08 was detected only in NIR platform. The difference between both could be due to selection of 100 lines for HPLC analysis, number of replication, and/or sample number for QTL analysis (Figure S5). An epistasis analysis was subsequently performed to evaluate the interaction between these QTL regions identified under different environments; however, no epistatic interaction was detected between the major QTL mapped on Chrs. 6 and 8 (data not shown).

As described, seed sample collections of the RIL population harvested in four different environments, years, and locations were quantified for the present study. Phenotypic variation analysis apparently showed the environmental effects on three seed composition traits studied (Figure S1), especially total oil and sucrose content of two parents and RILs quite varied among years and locations. It was possible that complex nature of quantitative traits coupled with great environment interactions resulted in the instability of QTL identified for seed composition traits in the present study (Table 1). The bin map gave a higher resolution (average 214 Kb) as compared to 3K‐SNP genetic map (average 1.2 Mb). In agreement with earlier studies in soybean (Qi *et al*., 2014; Xu *et al*., 2013), our results demonstrate that QTL mapping performed using skim‐WGRS significantly improves the mapping resolution compared to previous reports especially for protein and sucrose content. Xu *et al*. (2013) narrow down the soybean root‐knot nematode QTL up to 29.7 Kb, pinpointing three genes. It is well reported that in most cases, disease resistance is controlled by few major genes and therefore they successfully pinpoint three genes using WGRS. In another study, the skim‐WGRS of RILs was successfully utilized to improve QTL mapping resolution and identify the casual genes for salt tolerance in soybean (Qi *et al*., 2014; Xu *et al*., 2013). With all considerations, we are confident with the findings in our genetic mapping studies; however, we agree that NIR‐based data for sucrose content need to be validated by wet chemistry.

### Genome‐wide association mapping using skim‐WGS dataset

Genome‐wide association study has been proven to be useful for the identification of candidate loci in unrelated genotypes associated with numerous traits in crop plants, including soybean (Bandillo *et al*., 2015; Patil *et al*., 2016; Sonah *et al*., 2015; Vuong *et al*., 2015). Sonah *et al*. (2013) successfully demonstrated GWAS in diverse and biparental mapping population using genotyping‐by‐sequencing (GBS) approach. They evaluated several simple and complex traits, including oil and protein using GBS‐GWAS approach. In the present study, we utilized over 91 000 SNPs derived from the Williams 82 × PI 483460B population and performed GWAS analysis to support the QTL analysis and to verify major QTL (Figure S6). Consistency between GWAS and QTL mapping was observed for major QTL only and the minor QTL were below the GWAS significance threshold and this could be due to the controlled residual variances and the use of conservative statistical models: Bonferroni and permutations tests. A number of statistical approaches for declaring significance threshold have been developed including Bonferroni and permutations corrections. These methods correct the error rate by dividing the significance level at each locus by the number of tests and hence more conservative and reliable for declaring *P*‐value thresholds (Panagiotou and Ioannidis, 2011).

In agreement with earlier studies, GWAS enabled us to increase the resolution within QTL interval of major QTL (Mammadov *et al*., 2015; Sonah *et al*., 2015). Mammadov *et al*. (2015) used genetic linkage and GWAS (referred as hybrid mapping) for grey leaf spot disease in maize and dramatically increased the resolution within the confidence intervals. In our study, for protein, two significant loci were identified on Chr. 20, which can be correlated with QTL identified in BREC12 of bin map and all locations of 3K‐SNP QTL (Figure S6a). Similar to what we observed in our GWAS analysis, Sonah *et al*. (2015) also identified two genomic loci on Chr. 20. In the case of oil content, locus on Chr. 5 was identified using GWAS in the CR13 environment and it was consistent with QTL identified using 3K‐SNP‐based genetic map (Figure S6b). Compared to QTL mapping for oil, a new genomic locus on Chr. 18 was identified in GWAS. In the case of sucrose GWAS, a major locus was identified on Chr. 8 consistent with QTL mapping (Figure S6c). This is the first report of association and genetic mapping of seed sucrose content in soybean. The qSuc‐06 QTL were not observed in GWAS.

Subsequently, we performed GWAS analysis for a simple Mendelian trait (e.g. seed coat colour). The causal genes for seed coat colour were well known, and hence, this trait was used to validate our GWAS approach (Figure S7). Soybean seed coat colour is regulated by *I* locus on Chr. 8. The dominant forms (*I* and *i* ^*i*^) of the *I* locus inhibit pigmentation of the seed coat in a spatial manner resulting in a completely yellow seed (*I* allele) or a black seed (*i* allele) (Tuteja, 2007). For seed coat colour, as expected, a single region on Chr. 8 showed significant marker‐trait association with *P‐*value 5.17E‐10 (Figure S7).

### Candidate genes for seed composition traits

Traditional QTL analysis using a high‐density bin map identified several significant QTL for three seed composition traits. After the integration of bin QTL mapping and GWAS analysis, QTL with large effect (based on LOD score and −log10 value) and consistency in more than two environments were considered (Chrs. 2, ‐6, ‐8, ‐19, and ‐20) for gene mining with the soybean reference genome (Table S3). The gene ontology (GO) showed that majority of ‘biological_process’ was significantly enriched for fatty acid and carboxylic acid metabolism processes (Table S4). Furthermore, REVIGO web tool (http://revigo.irb.hr/) was used to visualize nonredundant GO term sets. The GO term of genes underlying consistent QTL was significantly enriched for fatty acid and carbohydrate metabolism (Table S4).

For protein and oil traits, the confidence interval QTL on Chr.20 that was flanked by bin_20_33975596 and bin_20_34027051 markers revealed 13 genes (Table S3). Among these, *Glyma.20G096700* encodes for Kelch motif family protein. This motif is known to be specifically enriched in acyl‐binding proteins associated with lipid‐transport protein and could change acyl‐CoA and TAG composition in canola (*Brassica napus*) seeds (Raboanatahiry *et al*., 2015). *Glyma.20g096900* encodes the eukaryotic carboxylate clamp‐TPR. In Arabidopsis, this gene was associated with Hsp90 proteins and performs key roles in signal transduction by regulating maturation, localization, stability, and protein interactions of a large number of signalling proteins. A homologue of Hsp90 gene in Arabidopsis (*AtHsp90‐1*) highly expresses in seed tissue (Prasad *et al*., 2010). *Glyma.20G097200* encodes mitochondrial pentatricopeptide repeat protein which functions in seed development and plant growth (Gutiérrez‐Marcos *et al*., 2007). Interestingly, two transcription factors (TFs), *Glyma.20G096100* encoding basic‐helix–loop–helix (bHLH) protein and *Glyma.20G096200* encoding high mobility group family protein, were identified. It has been reported that bHLH TF expresses at a higher level in mature seeds and associated with flux transport during soybean and maize seed development (Grimault *et al*., 2015; Jones *et al*., 2010). In Arabidopsis, bHLH TF complex activates expression of GLABRA2 in seed coat epidermis, which, in turn regulates seed oil content (Shi *et al*., 2012). Additionally, three genes with unknown function were identified (Table S3).

In the case of sucrose, genes harboured in the qSuc_06 and qSuc_08 QTL regions were taken into account for candidate gene mining. Several genes associated with carbohydrate metabolic pathway (*Glyma.06G218500*,* Glyma.06g221100*,* Glyma.08g098500*,* Glyma.08g109100*,* Glyma.08g111000*, and *Glyma.08g112100*) were identified (Table S3). Notably, two genes *Glyma.06g218500* and *Glyma.08g098500* responsible for sugar transporter protein were also identified. It has been well studied that sugar transporters (SUT and SWEET) play important role in transporting and unloading sugar molecules from source (leaf) into sink (seeds) tissues (Patil *et al*., 2015) and responsible for carbon partitioning (Baker *et al*., 2012). Gene *Glyma.08G109100* encodes UPD‐D‐glucuronic acid (UDG). In the haplotype analysis, this gene carries for nonsynonymous SNPs in several lines from 106 WGRS data set and associated with elevated sucrose concentration (Figure 5, Table S9). This enzyme directly competes with sucrose synthase and affects channelling carbohydrate between the target sucrose and other cell wall‐related residues. Mutation in UDG gene (*ugd2,3*) was found to be associated with increased concentration of glucose and sucrose in Arabidopsis (Reboul *et al*., 2011). Interestingly, the QTL qSuc_08 overlaps with a soybean domestication trait, for example seed coat colour, which comprises chalcone synthase loci (CHS1) (Figure 3). Gene *Glyma.08g111000* produces beta‐galactosidase enzyme involved in carbohydrate hydrolysis and found in close proximity to CHS I loci. Based on comparative proteomics analysis of seed coat colour, it has been reported that beta‐galactosidase metabolic proteins were up‐regulated during seed coat maturation and at the same time expression of sucrose‐binding protein was down‐regulated (Kim *et al*., 2013). The results suggested that seed coat colour plays an important role in nutrient transport to the developing embryo. Recently, Dobbels *et al*. (2017) identified a structural variation using comparative genome hybridization assay for a single gene (*Glyma.08g084300*) on Chr. 8 in a mutant line developed from a fast neutron mutagenized population. The mutant line showed elevated sucrose content (~9%) compared to its parent background M92‐220 (5%) (Dobbels *et al*., 2017). Further, they reported that this gene affected the translocation event and could be related to sucrose content. This gene encodes 3‐ketoacyl‐acyl carrier protein synthase I (KAS I) and is involved in one of the conversation steps between sucrose and fatty acids. The KAS I gene was 1.1 Mb from the QTL we identified (bin_8_7951579) in our study. In addition to metabolic pathway genes, few TF genes were also identified in this study. However, their role in seed development or soybean seed sucrose is unknown. If these fatty acid‐related genes are co‐regulated with sucrose, the speculation would be that the amount of sucrose being converted to fatty acids is decreasing, thus accumulating more sucrose in the seed. However, this finding warrants further wet chemistry analysis of the mutant seeds.

### Domestication traits associated with seed composition QTL

Cultivated soybean was domesticated in Asia from divergent populations of wild soybean, about 5000 years ago (Hyten *et al*., 2006; Kim *et al*., 2012). During the domestication processes, soybean has undergone significant phenotypic changes, including plant architecture, seed size, seed colour, and other agronomically important traits, such as seed composition and yield (Valliyodan *et al*., 2016). The QTL reported in this study coincided with the domestication traits, such as seed coat colour (*I* locus—Chr. 8), and maturity gene locus (E1—Chr. 6 and E4—Chr.20) (Figure 3). The seed coat colour is the most dramatically modified element under the domestication and artificial selection process (Valliyodan *et al*., 2016; Zhou *et al*., 2015) and facilitates the segregation and identities preservation of seeds with enhanced compositional traits (Tuteja *et al*., 2009).

### Haplotype analysis and candidate gene mining of significant QTL

**Figure 4:**
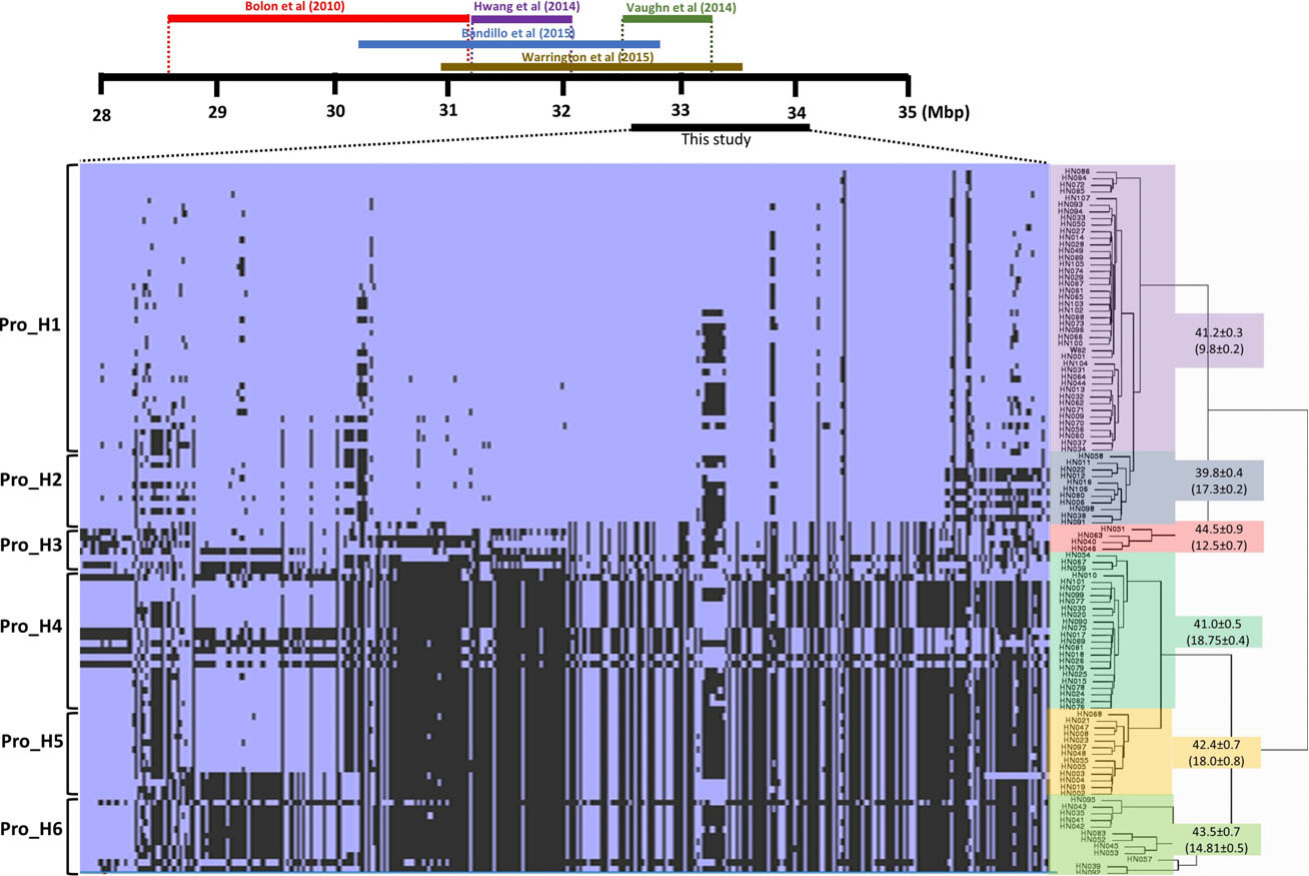
Haplotype analysis using WGRS data: analysis of 106 soybean WGS data (Valliyodan *et al*., 2016) identified different haplotypes underlying the major seed protein and oil QTL on Chr. 20. The QTL identified in previous studies are overlaid with different colour bars. Protein and oil content (parenthesis) mentioned next to each phylogenetic cluster. Blue colour represents reference allele (W82) and black represents alternate allele.

Although multiple QTL were identified (Table 1), haplotype analysis was solely performed on major significant QTL, qSuc_08, and qPro_20 for sucrose and protein/oil content, respectively. The 106 soybean lines sequenced at 15× genome coverage were utilized to infer SNP clustering and haplotype analysis (Valliyodan *et al*., 2016). To observe phylogenetic clustering, multisampled SNPs for protein/oil (Gm20: 32.5–34.1 Mbp) and sucrose (Gm08: 7546707–8631775) were extracted from the WGRS data. The hierarchical clustering of qPro_20 region identified two distinct clusters which were further divided into subclusters of three (Figure 4, Table S5). This region overlaps with earlier GWAS (Bandillo *et al*., 2015; Hwang *et al*., 2014; Vaughn *et al*., 2014) and QTL (Bolon *et al*., 2010; Warrington *et al*., 2015) studies (marked with coloured bars). In this region, approximately 4100 SNP were identified. Further we extracted the large effect SNP and identified 19 nonsynonymous SNP underlying qPro_20 QTL wherein the amino acid change in Glyma.20g096100 (R145H), Glyma.20g096800 (S12F, P19L, and C172F), and Glyma.20g097400 (E97V) was predicted as deleterious mutation based on Protein Variation Effect Analyzer (http://provean.jcvi.org/index.php) (Tables S7 and S8). The Glyma.20g096100 encodes bHLH TFs and Glyma.20g097400 encodes Homeobox TF and up‐regulated in during flower and seed tissue development (soybean eFP browser; http://bar.utoronto.ca/) and hence could be considered as potential genes involved in storage protein accumulation. The *Glyma.20g096800* contains three large effect SNPs; however, its function is not known. Recently, Deshmukh *et al*. (2015) have experimentally proved the effect predicted with similar approach using site‐directed mutagenesis. In this study, they identify the role of aquaporin transporter gene for silicon transport in plants (Deshmukh *et al*., 2015). Analysis of variants (ANOVA) based on seed protein content, Pro_H3 (44.48 ± 2.1%) and Pro_H6 (43.49% ± 1.0), showed significantly higher protein content (*P *=* *0.007; *P *<* *0.05). While the Pro_H3 lines are *G. soja* lines, a majority of the Pro_H6 lines are South Korean accessions. When the elite genotypes from South Korea (44.06 ± 1.8%) compared with the USA (41.12 ± 1.3%) and China (42.10 ± 1.4%), Korean elite lines showed a significantly higher protein. It has been reported that geographical origin and maturity groups are the principal determinants of population structure and phenotypic variation and accessions from Korea form a unique population structure compared with Chinese and US accessions (Patil *et al*., 2017b). Furthermore, GWAS for protein content based on geographical origin identified the strongest association on Chr. 20 in Korean and Japanese subpopulations, whereas these regions were not prevalent in the US and South‐East Asia (Vaughn *et al*. 2014; Bandillo *et al*., 2015). These observations suggest that breeding strategies for higher protein content can be attributed to a breeding focus on soy protein food products. Although *G. soja* carries unique alleles, the underutilization of wild accessions for seed traits could be due to linkage drag on favourable agronomic characteristics (Patil *et al*., 2017b).

The qPro_20 identified in this study overlapped with the QTL region previously reported in independent studies, suggesting that traditional linkage mapping integrated with haplotype analysis could infer allelic variation within QTL region. Interestingly, lines belong to Pro_H1 and Pro_H2 contents relatively lower protein, and the majority of lines in this haplotype are elite cultivars developed in the US. The US soybean germplasm has narrow genetic diversity that resulted from a severe population bottleneck and only 17 North American Ancestor lines contributed 86% of modern US cultivars (Hyten *et al*., 2006). This observation indicated that most of the commercial soybean cultivars in the US are fixed for the low protein allele at qPro_20 QTL. However, introgression of desired high protein allele from Pro_H6 or Pro_H3 would enhance seed protein content.

**Figure 5:**
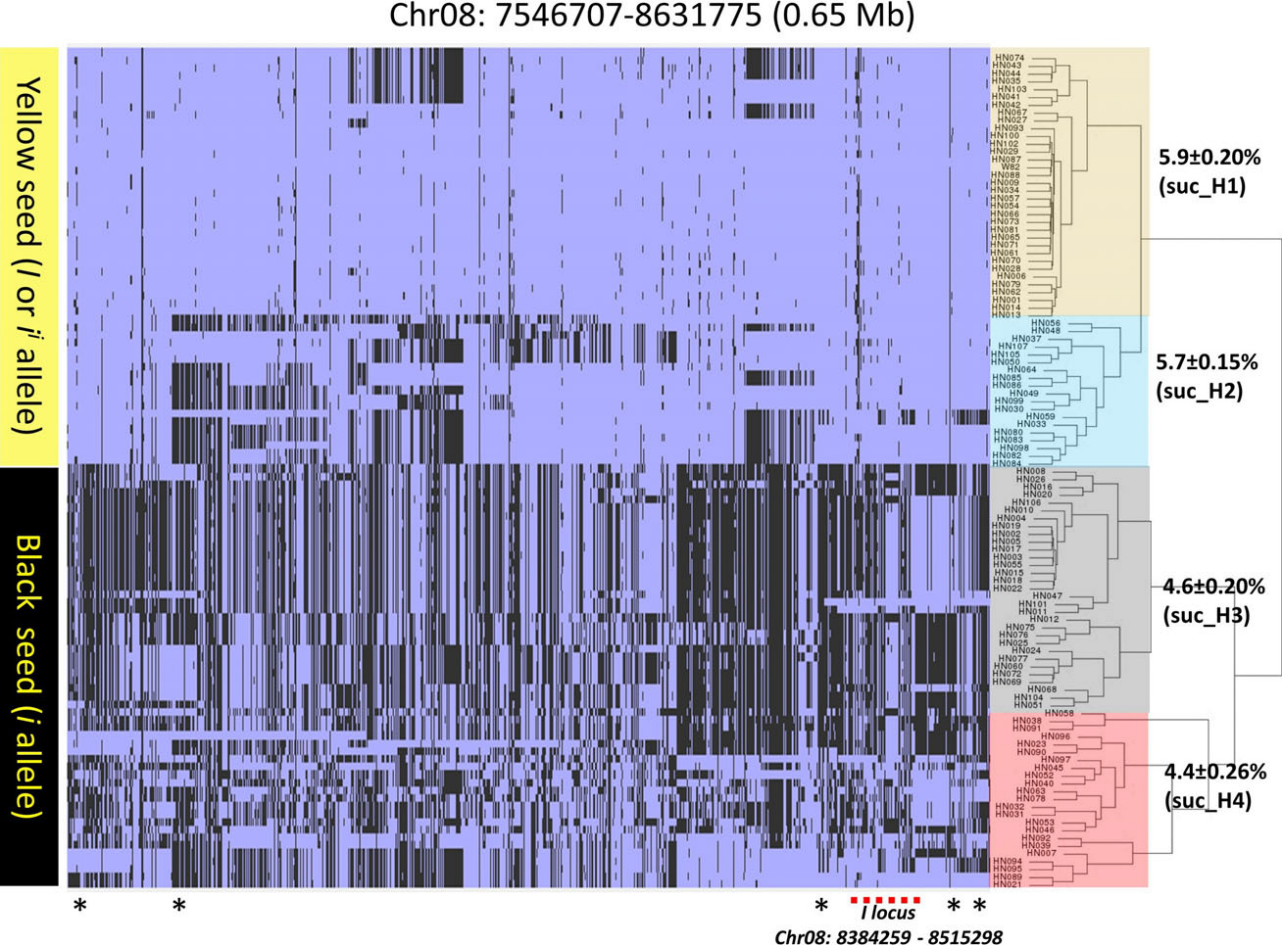
Haplotype analysis and allelic variation on Chr.8 suc‐QTL using WGRS data. Analysis of 106 soybean WGS data (Valliyodan *et al*., 2016) identified distinct haplotypes underlying the major seed sucrose and QTL on Chr. 8. The *I* locus associated with seed coat colour is marked with a dotted red line. Genes associated with carbohydrate biosynthesis are marked with a black asterisk. Sucrose content mentioned next to each phylogenetic cluster. Blue colour represents reference allele (W82) and black represents alternate allele. Statistical significance was assessed using unpaired two‐tailed Student's *t* tests (*P *> 0.05).

The allelic variation for the qSuc_08 QTL was examined to evaluate the difference in sucrose content (Figure 5). The hierarchical clustering identified two distinct haplotypes of 53 and 54 lines, which were further divided into subcluster suc_H1‐suc_H2 and suc_H3‐suc_H4 (Figure 5, Table S6). The suc_H1 and suc_H2 showed relatively higher sucrose content when compared to suc_H3 and suc_H4. Furthermore, we identified 131 nonsynonymous SNP in the genes underlying qSuc_08 QTL and were associated with sucrose content haplotypes. About 33 amino acid changes were predicted as deleterious mutations in 22 genes and may impact the biological function of the protein (Tables S9 and S10). The majority of yellow seed coat lines (suc_H1 and suc_H2) comprise elite soybean lines, supporting the common hypothesis that the seed coat colour (*I* locus) is the most dramatically modified trait under the domestication and artificial selection process (Tuteja *et al*., 2009). Importantly, our analysis identified all known alleles (*I*,* i* ^*i*^ and *i*) of *I* locus containing a cluster of chalcone synthase genes involved in an anthocyanin pathway. The other two loci (*T* and *R*) associated with seed coat colour were not observed in our analysis and it could be due to the mapping parents used in present study carries only *I* locus associated with yellow/black seed coat colour whereas *T* and *R* locus black/brown seed coat colour (Gillman *et al*., 2011; Song *et al*., 2016; Toda *et al*., 2002).

## Summary

In summary, an interspecific biparental population was developed from a cultivated soybean cultivar, Williams 82, and a wild accession, PI 483460B, for a genetic characterization of seed composition traits. The analysis successfully identified and mapped significant QTL for seed protein, oil, and sucrose content using Illumina BeadChip and skim‐WGS platforms. With a large volume of SNP markers derived from the skim‐WGS work, a very high‐resolution bin map was developed and showed co‐linearity of the bin map with physical map. The bin‐based genetic mapping when compared with 3K‐SNP identified narrow QTL intervals resulting in fewer putative candidate genes that were functionally annotated with the trait. More importantly, a novel QTL for sucrose content were identified and underlies several candidate genes associated with sugar transport mechanism. The QTL identified for protein, oil, and sucrose coincided with the domestication traits, such as plant maturity (*E1* and *E4* loci) and seed coat colour (*I* locus), supporting the common hypothesis of artificial selection for seed composition traits. In addition to linkage mapping, GWAS and haplotype analysis supported the consistent QTL across the environments and inferred the allelic variation within identified QTL, respectively. In terms of protein and oil, the three major haplotypes on qPro_20 showed significant association with protein content and distinguished North American cultivars from Korean lines and wild soybean. These QTL could be potential target for trait introgression and molecular marker development towards the improvement of protein meal quality and sucrose content.

## Materials and methods

### Plant materials

An interspecific population of RILs was derived from a cross of cultivar Williams 82 (Bernard and Cremeens, 1988) and PI 483460B. Cultivar Williams 82 was the first soybean reference genome, and PI 483460B is a wild soybean (*G. soja*) accession originated from China (http://www.ars-grin.gov). These parental lines significantly differ in seed composition traits, seed coat colour, and seed size (Table S1). They both are also classified as maturity group (MG) III. Field crossing to produce F_1_ hybrids was made at the Delta Research Center, the University of Missouri, Portageville, MO. Following the verification of F_1_ true hybridization, F_2_ seed generation was advanced in the soybean nursery in Costa Rica. The mapping population of 188 F_7:8_ RILs was developed using single seed descent method. These RILs and the parents were grown in four environments, summer of 2012, 2013, and 2014 in the Bradford Research and Education Center (BREC), Columbia, Missouri, and spring of 2013 in the soybean nursery in Costa Rica (CR).

### DNA preparation, genotyping, SNP development

Genomic DNA samples were isolated from pooled leaf tissue of five seedlings of each F_7:8_ RIL and their parents using an automated Autogen 960 system and the CTAB protocol (AutoGen Inc., Holliston, MA) with minor modifications as previously described (Vuong *et al*., 2010). DNA samples were quantified by PicoGreen, and about 200–400 ng DNA/sample was analysed using the Illumina Infinium assays, following the protocol described by Illumina Inc. (San Diego, CA). Briefly, the DNA samples were first isothermally amplified overnight throughout the whole genome. The products were then enzymatically fragmented using restriction enzymes. The fragmented DNAs were precipitated and resuspended in buffer to prepare them for hybridization to the chip. The BeadChips were prepared for hybridization in capillary flow‐through chambers provided by Illumina Inc. The samples were subsequently loaded to BeadChips and incubated overnight, during which the fragmented amplified DNA hybridizes to the locus‐specific 50‐mer oligos that had been covalently linked to beads in the array synthesis process. After hybridization, allelic specificity was conferred by a single‐base extension, followed by fluorescence stains to amplify the signal. After drying, the bead's fluorescence intensities were detected by Illumina BeadArray Reader (Illumina Inc.). SNP calling was automated using the Genome Studio program, with manual modifications when needed (Infinium^®^ II Assay Workflow, Pub. No. 370‐2006‐027 07Dec06).

A total of 16 469 SNP markers were included in the final Illumina Infinium BeadChip for genotyping. These SNP set included 7113 SNPs developed at the Soybean Genomics and Improvement Laboratory, USDA‐ARS, Beltsville, MD, and 9356 SNPs developed at the National Center for Soybean Biotechnology, the University of Missouri, MO.

### Construction of BeadChip‐based (3K) genetic linkage map

A genetic linkage map was constructed for the Williams 82 × PI 483460B population using MSTmap as previously described (Wu *et al*., 2008). Based on the population size and the number of markers in a genotypic data set, the parameters specified for the MSTmap software were as follows: Kosambi, *P*‐value cut‐off: 1.0E‐13 for Genetic mapping function; 2 for No mapping size threshold; 10 cM for No mapping distance threshold; and 0.4 for No mapping missing threshold. The map quality was manually improved by removing markers with significant segregation distortion and misplaced markers compared to the physical map of the Williams 82 reference genome. Of 9356 SNPs in the Infinium chips, over 6000 markers were found to be polymorphic between the two parents and were incorporated into linkage analysis. The total genetic linkage map distance was 2925 cM. The number of SNP markers and length of each chromosome is presented in Table S2.

### Skim whole‐genome sequencing and SNP calling

One hundred and eighty‐eight F_7:8_‐derived RILs and two parental lines were grown in a greenhouse of the University of Missouri. Two‐week‐old plantlet leaves were collected to extract genomic DNA using the standard CTAB method. Approximately 50 ng DNA/sample was shipped to Beijing Genomics Institute (BGI, http://www.bgi.com, Shenzhen, China) for library construction and Illumina sequencing. Briefly, paired‐end (PE) sequencing libraries with an insert size of approximately 125 bp were constructed. The reads obtained were filtered to remove low‐quality bases and used for SNP identification. Initially, the reads from the parental genotypes (PI 483460B) were aligned to the reference soybean genome (W82.a2.v1) using SOAP3 (Liu *et al*., 2012). Uniquely mapped reads were considered for SNP calling using SGSAutoSNP software (Lorenc *et al*., 2012) with default parameters. Similarly, the low‐quality reads obtained from RILs were filtered out and SNPs were identified from the remaining reads. The identified SNPs were then filtered with a minor allele frequency (MAF) cut‐off of 0.2, and lines with ≥5% missing data were also excluded. SNPs were scored as ‘A’ and ‘B’ representing alleles from the two parents, Williams 82 and PI 483460B, respectively.

### Identification of recombination breakpoints and construction of bin and linkage map

An 18‐bp sliding window approach was used for identification of true recombination breakpoints in RIL population (Huang *et al*., 2009b). The breakpoints were determined based on the ratio of alleles in the window using Perl script modified as per the soybean genome coordinates. Briefly, for each individual, the ratio of ‘A’ and ‘B’ alleles within the window was determined and windows with 12 or more alleles from either parent were considered homozygous for an individual. The recombination breakpoints were then determined as a transition from one genotype to other. Further, the recombination breakpoints identified from all the individual RILs were combined and compared over the 100‐Kb interval. The intervals lacking recombination in the entire population were merged and considered as a single bin.

The bins were used as genetic markers for the construction of a linkage map. Map construction was carried out using QTL IciMapping Version 3.3 software (Meng *et al*., 2015). The REcombination Counting and ORDering (RECORD) algorithm was used for marker ordering. Sum of adjacent criterion ripple was performed to confirm the marker order. The marker order and their positions on genetic and physical map were visualized using Strudel V. 1.12.03.20 (Bayer *et al*., 2011).

### Seed composition trait phenotyping

Approximately 5–6 g of soybean seed was finely ground using Mini‐Mill (Thomas Wiley, Swedesboro, NJ) fitted with 20‐mesh screen and used to quantify seed protein, oil, and sucrose content using NIR spectroscopy. The spectrophotometer was a FOSS NIR System 6500. Two individual samples from each replication of each location were analysed at regular interval to confirm repeatability of measurements (Baianu *et al*., 2012). For the correlation analysis, seed samples of 100 RILs from the experiment grown at the BREC in 2013 and two parents were evaluated on HPLC (Agilent Technologies, Santa Ana, CA) as described by Valliyodan *et al*. (2015).

### Data analysis

Phenotypic data of seed protein, oil, and sucrose content were subjected to an ANOVA using the PROC GLM mixed model of SAS version 9.2 (SAS Institute, Cary, North Carolina). The linear statistical model contains the effects of environment, replication within the environment, genotype, and environment × genotype interaction. Environments were considered as fixed effects while genotypes and environment × genotype interaction were considered as random effects. Replication within environment was used as the denominator of the *F*‐value of the environment. The residual mean square was used to test genotype and genotype × environment interaction. Broad‐sense heritability (H2) of each trait was estimated on an entry mean basis following Nyquist and Baker (1991):

H2=(s2g)/[s2g+(s2ge/e)+(s2/re)],

where s2g is genetic variance, s2 ge is genotype × environment variance, s2 is error variance, r is number of replications, and e is the number of environments. Phenotypic correlations were determined using the PROC CORR procedure of SAS. Genetic correlations were calculated using the following formula (Falconer *et al*., 1996):

rG=Covxy/(s2xs2y)1/2,

where rG represents genetic correlation, Cov represents genetic covariance, x represents the first trait, y represents the second trait, and s2 is genetic variance. Cross‐products between traits were generated using PROC GLM procedure of SAS with MANOVA (multivariate analysis of variance) option.

### QTL analysis

A comprehensive approach for QTL analysis, including interval mapping, cofactor selection, genome‐wide permutation test, and multi‐QTL method, to detect and map significant QTL was performed using the program MapQTL 5.0 (Van Ooijen, 2004) as previously described (Vuong *et al*., 2010). A multivariate ANOVA model in SAS (SAS Institute, Cary, NY) was used to estimate the total phenotypic variation explained by the significant QTL. The prediction of epistatic interactions between significant QTL was performed using the computer program QTLNetwork 2.0 (Yang *et al*., 2007) with a mixed model. Significance levels for the genome scans for candidate intervals, QTL detection, and effects were set at 0.05, 0.001, and 0.001, respectively. The chromosomes with LOD plots were subsequently created using the MapChart 2.2 program (Voorrips, 2002) based on the outputs from MapQTL 5.0. The identified QTL were designated as qPro, qOil, and qSuc for protein, oil, and sucrose content followed by chromosome number.

### Genome‐wide association analyses of oil, protein, sucrose, and seed coat colour

A total of 91 342 SNPs (MAF < 0.05) were used to implement association analyses with a compressed mixed linear (MLM) model in GAPIT (Lipka *et al*., 2012). The population structure was accounted for by principle component analysis, and the kinship matrix was calculated using VanRaden method (K) to determine relatedness among individuals. The Bonferroni method at α ≤ 0.05 (corresponding to *P* ≤ 1.1 × 10^−6^) was used as the threshold to determine significant association (Holm, 1979).

## Conflict of interest

The authors declare no conflict of interest.

## Author contributions

GP conducted the QTL analysis, association mapping, haplotype analysis, data interpretation, and manuscript writing. TDV constructed linkage map, oversaw development of the population and phenotyping, and contributed to analysis and manuscript editing. SKale performed SNP calling, bin mapping, and contributed in manuscript writing. RD and CZ performed linkage mapping, data analysis, and interpretation. XW, YB, FL, and SK constructed SNP chip‐array. BV provided whole genome sequencing data. DY oversaw seed harvesting, packaging, and phenotyping. RKV provided computational resources for NGS data analysis and edited manuscript. GJS developed RIL population. HTN conceived the study and edited the manuscript. All authors read and approved the final manuscript.

## Supporting information


**Figure S1** Distributions of (i) protein, (ii) oil and (iii) seed sucrose content evaluated in the recombinant inbred lines of the Williams 82 × PI 483460B population grown in different environments. (a) At the Bradford Farm Education and Research Center (BREC), University of Missouri (MU), in summer of 2012; (b) at the BREC, MU, in summer of 2013; (c) at the BREC, MU, in summer of 2014; and (d) in the soybean nursery in Costa Rica in 2013.

**Figure S2** (a) Correlation between sucrose content estimated using HPLC and NIR platform. (b) Non‐linear correlation between NIR and HPLC platform (*n* = 100; environment: BREC14).

**Figure S3** (a) Distribution of SNPs in the 20 soybean chromosomes. The *x*‐axis represents the physical distance along each chromosome, split into 50 kb windows. The different color marks the SNP density in that particular region. (b) Genetic linkage map constructed in the Williams 82 × PI 483460B population using 4070 bins markers.

**Figure S4** Correlation between bin genetic map with a physical map. Red and green vertical lines represent bin linkage map and physical map respectively.

**Figure S5** QTL analysis on subset of 100 samples (BREC13) phenotyped with (a) NIR and (b) HPLC platform.

**Figure S6** Manhattan plots of GWAS for (a) protein, (b) oil and, (c) sucrose, in WPB RIL population using >91 K SNP dataset. Negative log_10_‐transformed *P* values of SNPs from genome‐wide scan using EMMAX model including kinship and population structure are plotted against positions on each of the 20 chromosomes.

**Figure S7** Manhattan plots of GWAS for seed coat color, in WPB RIL population using >91 K SNP dataset. Negative log_10_‐transformed *P* values of SNPs from genome‐wide scan using EMMAX model including kinship and population structure are plotted against positions on each of the 20 chromosomes.

**Table S1** (a) Mean, range, statistics, and heritability of seed protein, oil, and sucrose content (%) evaluated in F7:8 recombinant inbred lines (RILs) derived from an inter‐specific population and two parental lines, Williams 82 (*G. max*) and PI 483460B (*G. soja*). The RILs were grown in different field environments of years and locations. (b) Pearson correlation coefficients (*r* ^2^) and probability (Pr., in a second italicized line) among seed oil, protein, and sucrose content evaluated in F7:8 recombinant inbred lines (RILs) of an inter‐specific mapping population, Williams 82 (*G. max*) × PI 483460B (*G. soja*). This population was grown in different field environments of years and locations.
**Table S2** Distribution of SNPs, recombination bins and markers mapped on soybean chromosomes/linkage groups. (A) Skim‐WGS; (B) 3K‐SNP.
**Table S3** Identification of candidate genes underlying oil, protein and sucrose QTL.
**Table S4** Gene Ontology enrichment.
**Table S5** (A) Identification of allelic variation associated with protein/oil content, geographic origin, maturity, and group. (B) Analysis of variance for protein and oil haplotypes.
**Table S6** (A) Identification of allelic variation associated with sucrose content and seed coat color. (B) Analysis of variance for Sucrose haplotypes.
**Table S7** Identification on non‐synonymous SNPs underlying qPro_20 QTL.
**Table S8** Details of effects predicted for the amino acid changes observed naturally among soybean cultivars for the genes underlying seed protein QTL.
**Table S9** Identification on non‐synonymous SNPs underlying qSuc_08 QTL.
**Table S10** Details of effects predicted for the amino acid changes observed naturally among soybean cultivars for the genes underlying seed sucrose QTL.
