# Supplementary figures and images for: Dissecting genomic hotspots underlying seed protein, oil, and sucrose content in an interspecific mapping population of soybean using high‐density linkage mapping
Source: Plant Biotechnol J. 2018 May 16;16(11):1939–53. doi: 10.1111/pbi.12929 (PMC6181215; doi:10.1111/pbi.12929)

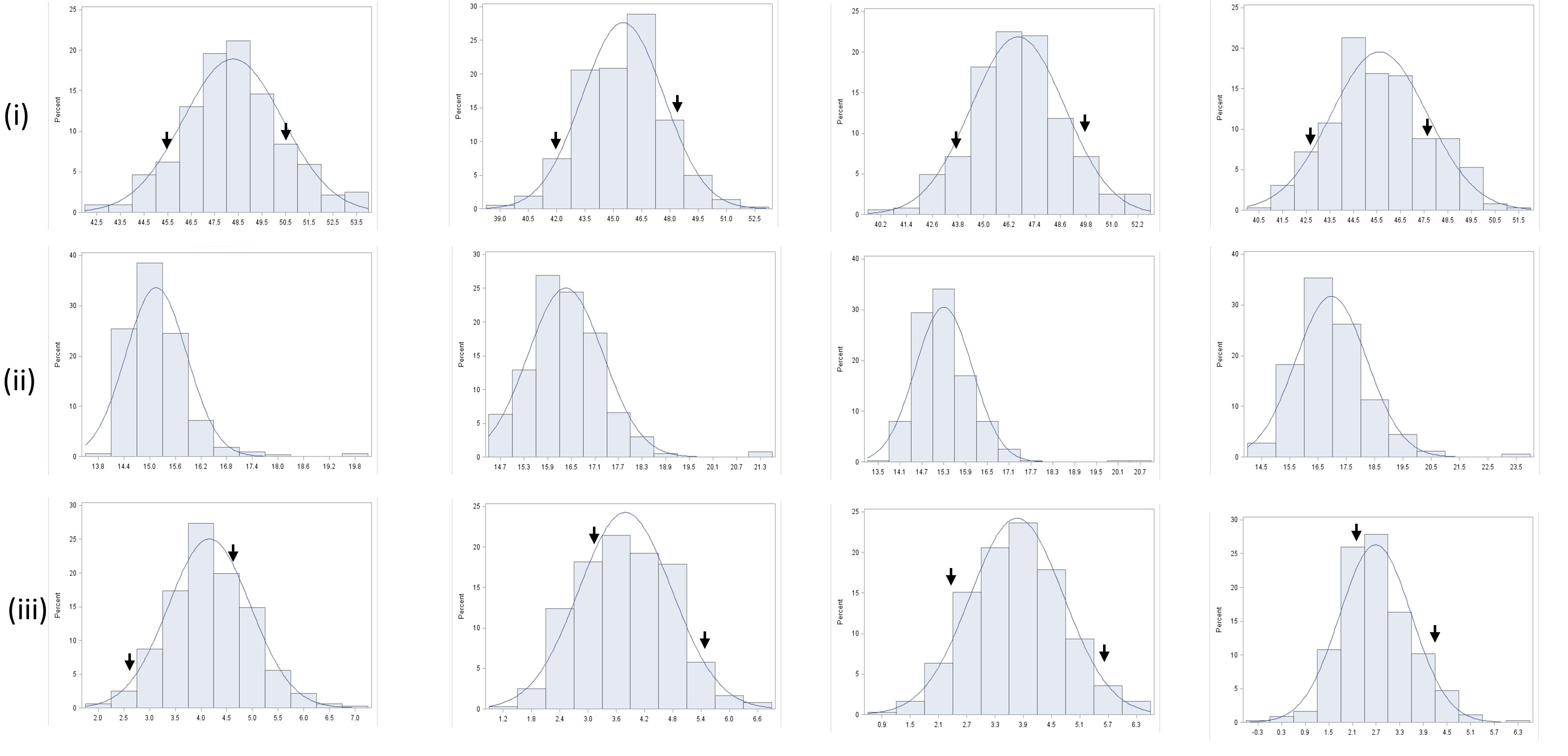

Supplement: Supplementary file 1 — Figure S1 Distributions of (i) protein, (ii) oil and (iii) seed sucrose content evaluated in the recombinant inbred lines of the Williams 82 × PI 483460B population grown in different environments. (a) At the Bradford Farm Education and Research Center (BREC), University of Missouri (MU), in summer of 2012; (b) at the BREC, MU, in summer of 2013; (c) at the BREC, MU, in summer of 2014; and (d) in the soybean nursery in Costa Rica in 2013. [file PBI-16-1939-s007.jpg]

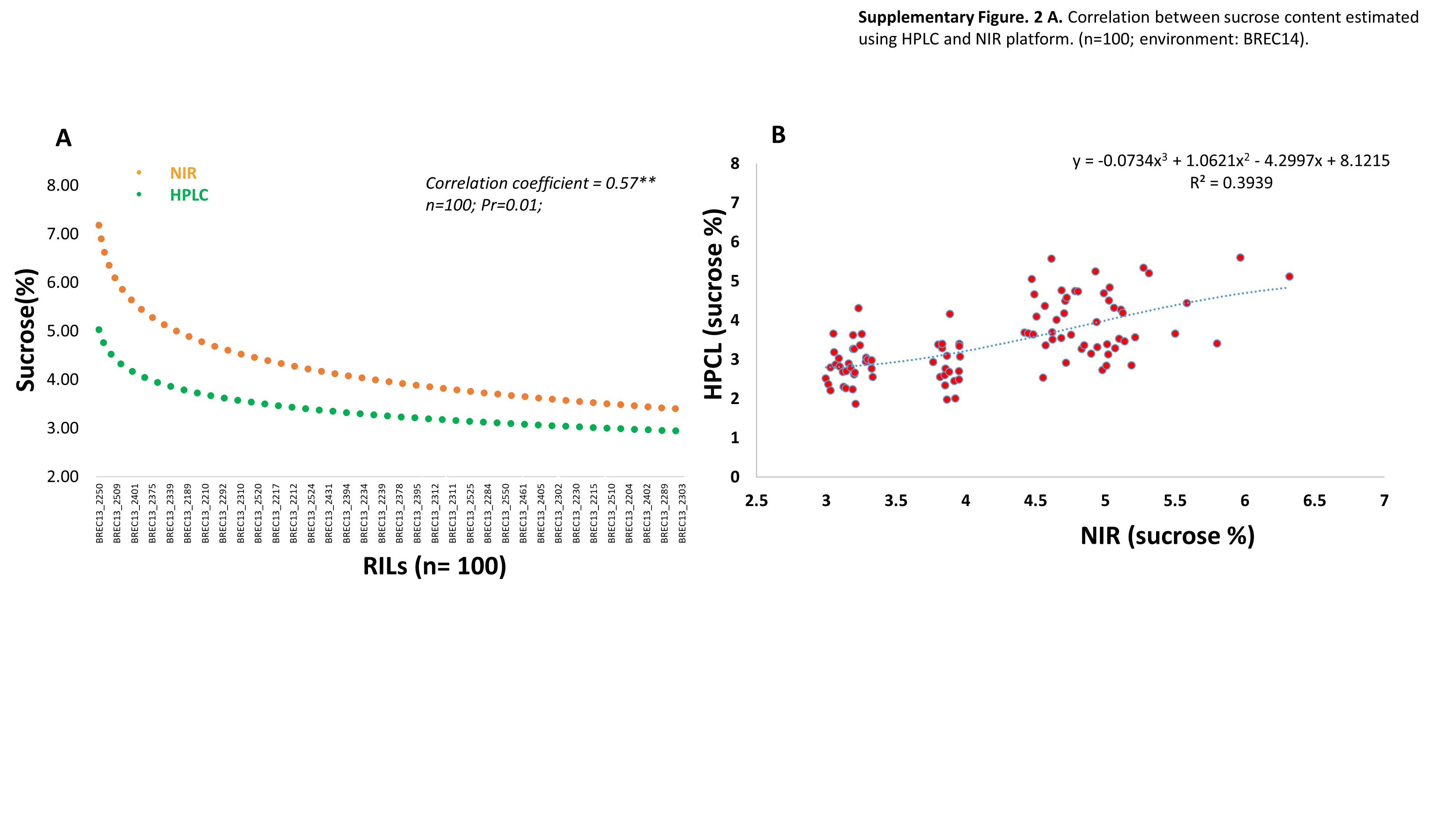

Supplement: Supplementary file 2 — Figure S2 (a) Correlation between sucrose content estimated using HPLC and NIR platform. (b) Non‐linear correlation between NIR and HPLC platform (n = 100; environment: BREC14). [file PBI-16-1939-s006.jpg]

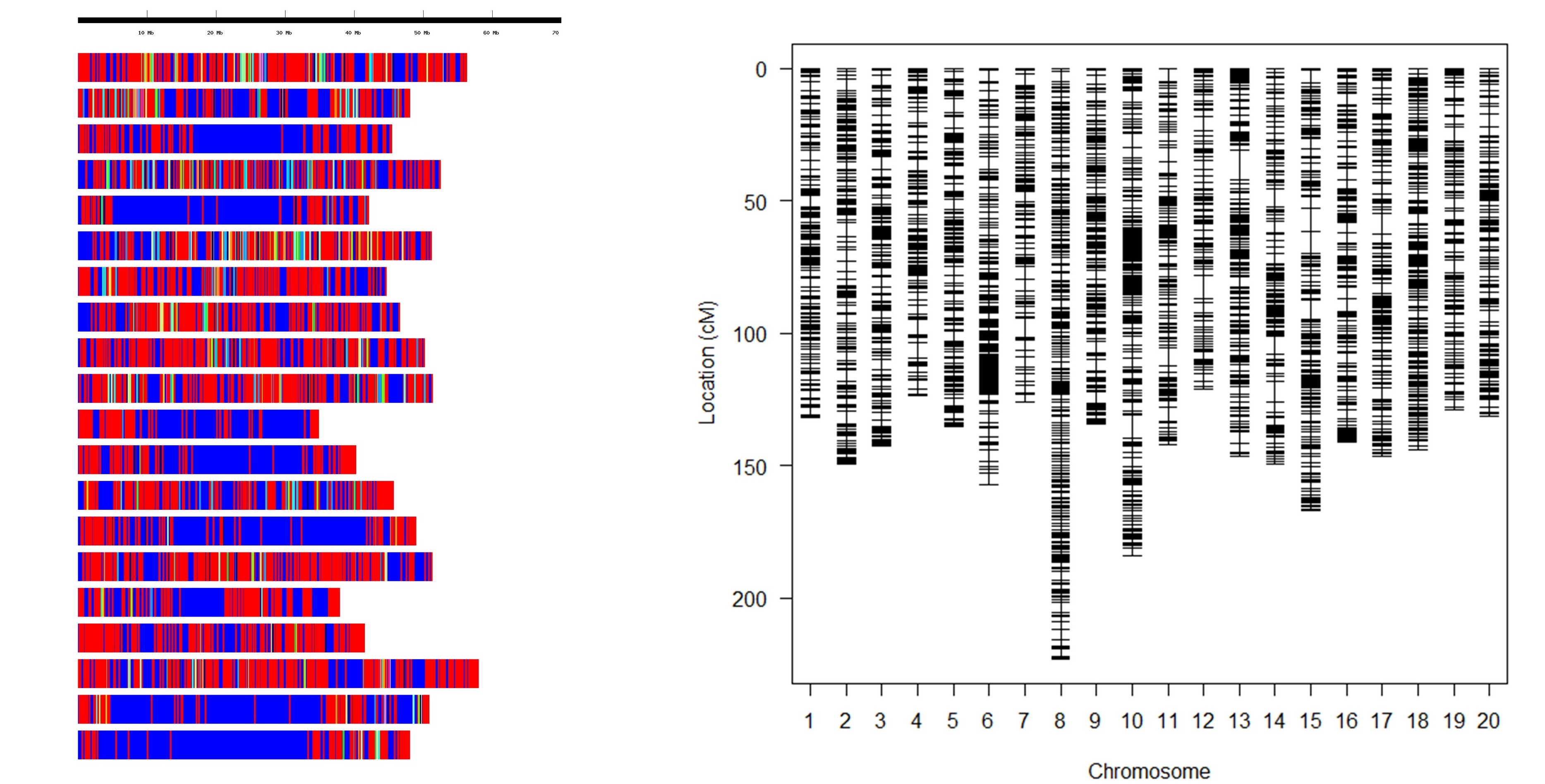

Supplement: Supplementary file 3 — Figure S3 (a) Distribution of SNPs in the 20 soybean chromosomes. The x‐axis represents the physical distance along each chromosome, split into 50 kb windows. The different color marks the SNP density in that particular region. (b) Genetic linkage map constructed in the Williams 82 × PI 483460B population using 4070 bins markers. [file PBI-16-1939-s005.jpg]

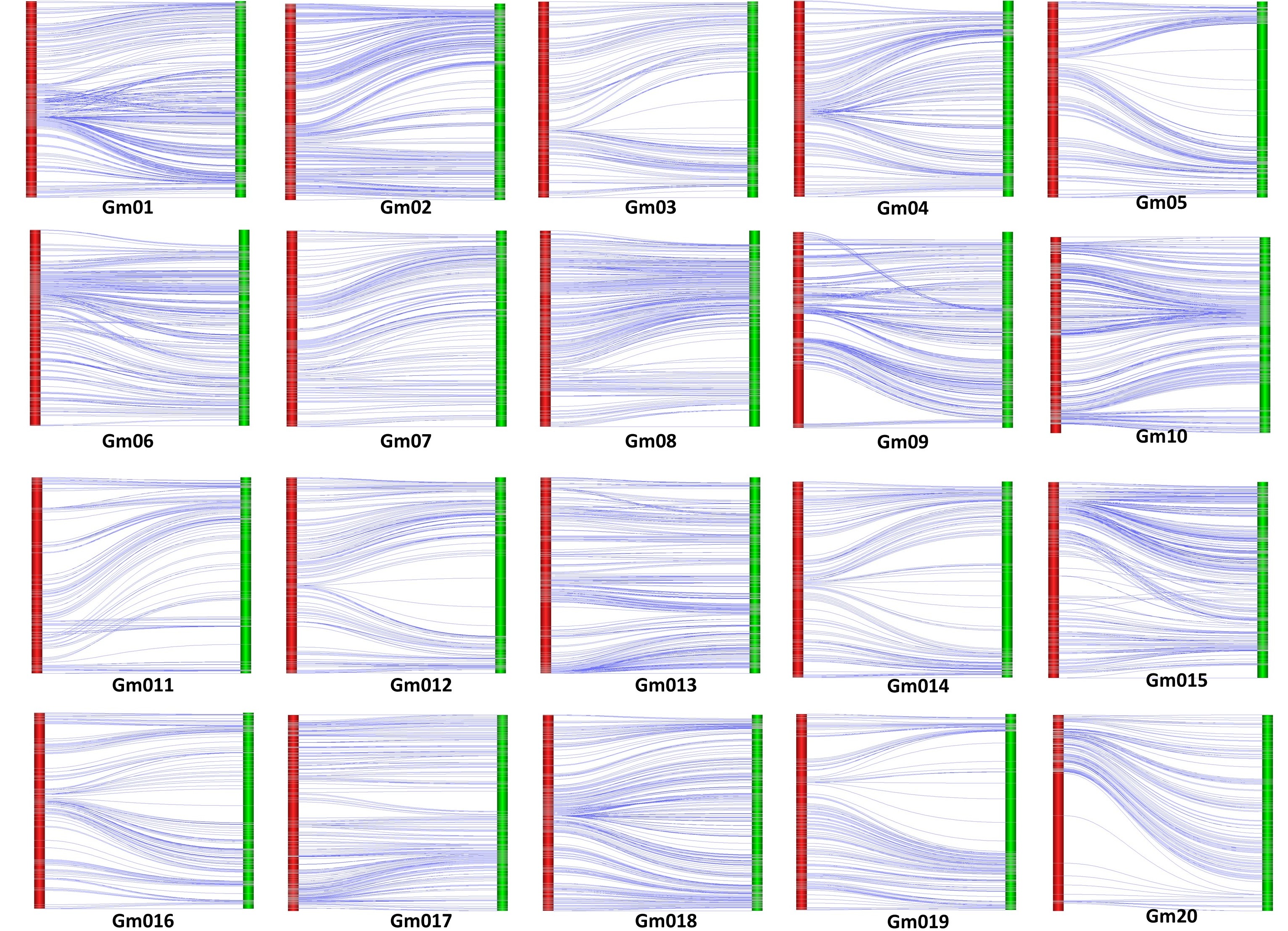

Supplement: Supplementary file 4 — Figure S4 Correlation between bin genetic map with a physical map. Red and green vertical lines represent bin linkage map and physical map respectively. [file PBI-16-1939-s004.jpg]

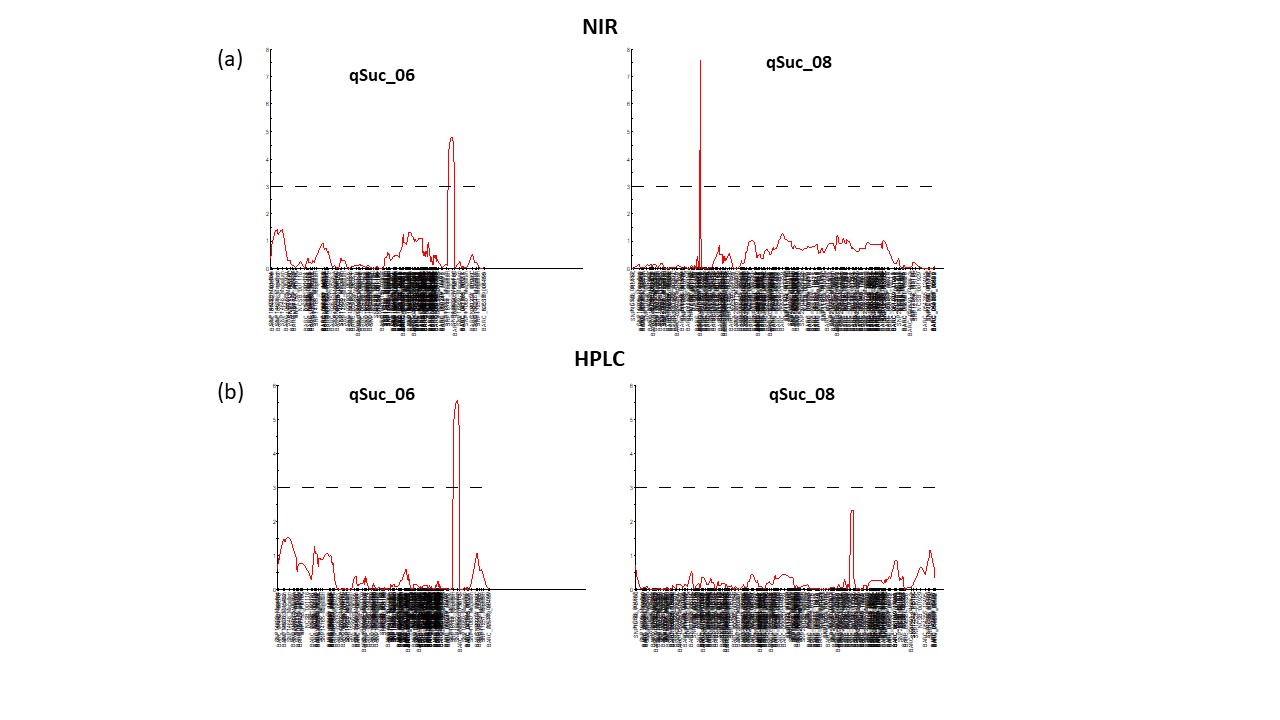

Supplement: Supplementary file 5 — Figure S5 QTL analysis on subset of 100 samples (BREC13) phenotyped with (a) NIR and (b) HPLC platform. [file PBI-16-1939-s008.jpg]

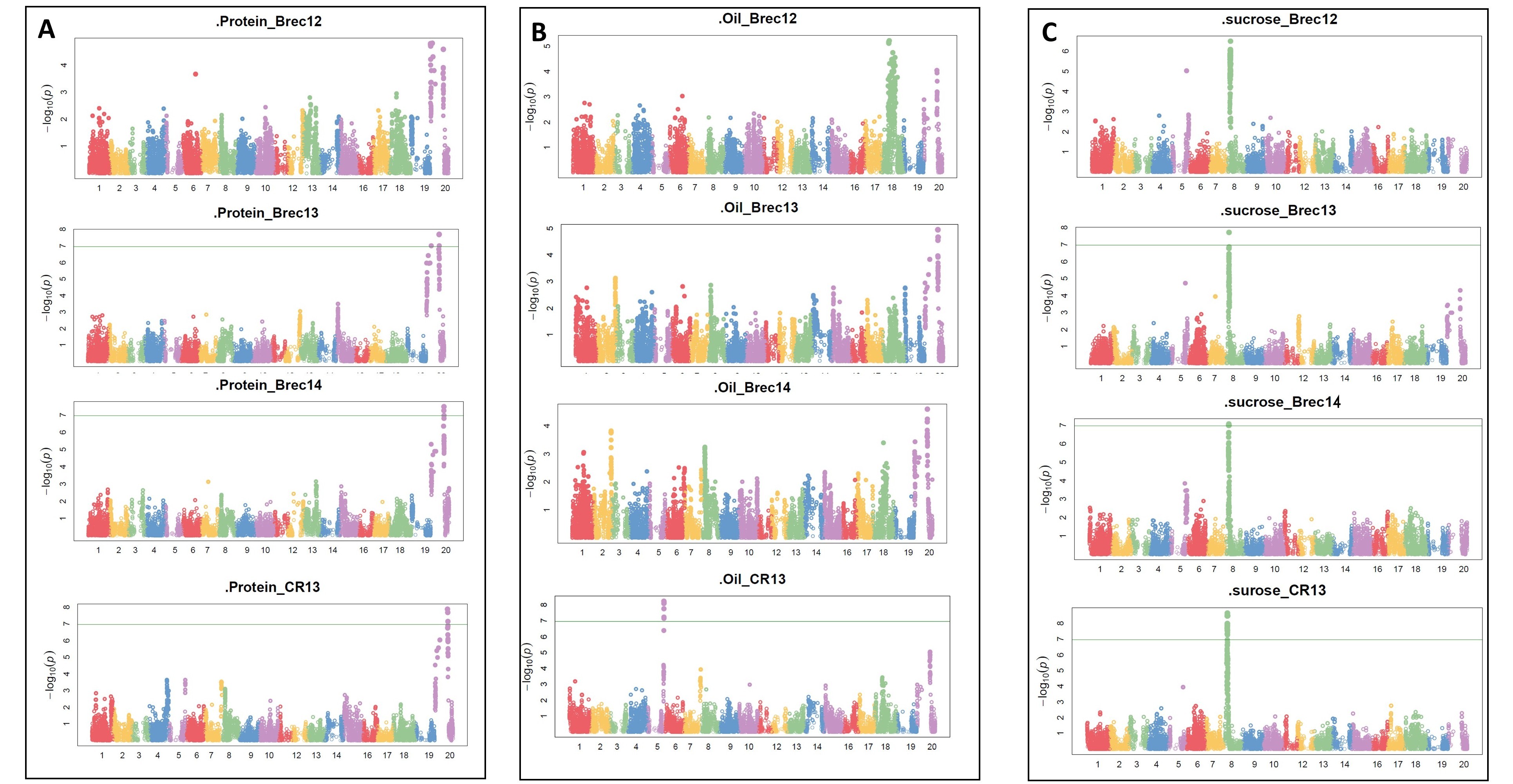

Supplement: Supplementary file 6 — Figure S6 Manhattan plots of GWAS for (a) protein, (b) oil and, (c) sucrose, in WPB RIL population using >91 K SNP dataset. Negative log10‐transformed P values of SNPs from genome‐wide scan using EMMAX model including kinship and population structure are plotted against positions on each of the 20 chromosomes. [file PBI-16-1939-s003.jpg]

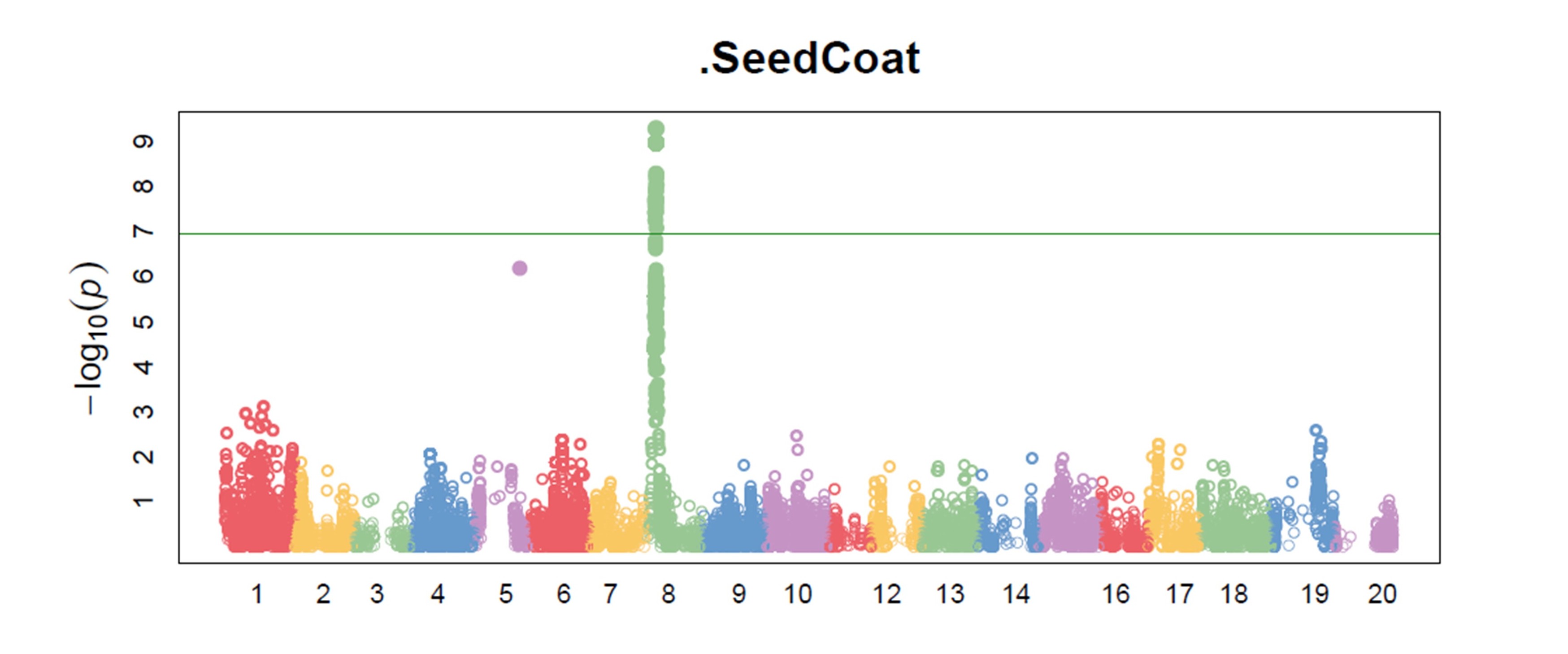

Supplement: Supplementary file 7 — Figure S7 Manhattan plots of GWAS for seed coat color, in WPB RIL population using >91 K SNP dataset. Negative log10‐transformed P values of SNPs from genome‐wide scan using EMMAX model including kinship and population structure are plotted against positions on each of the 20 chromosomes. [file PBI-16-1939-s002.jpg]
